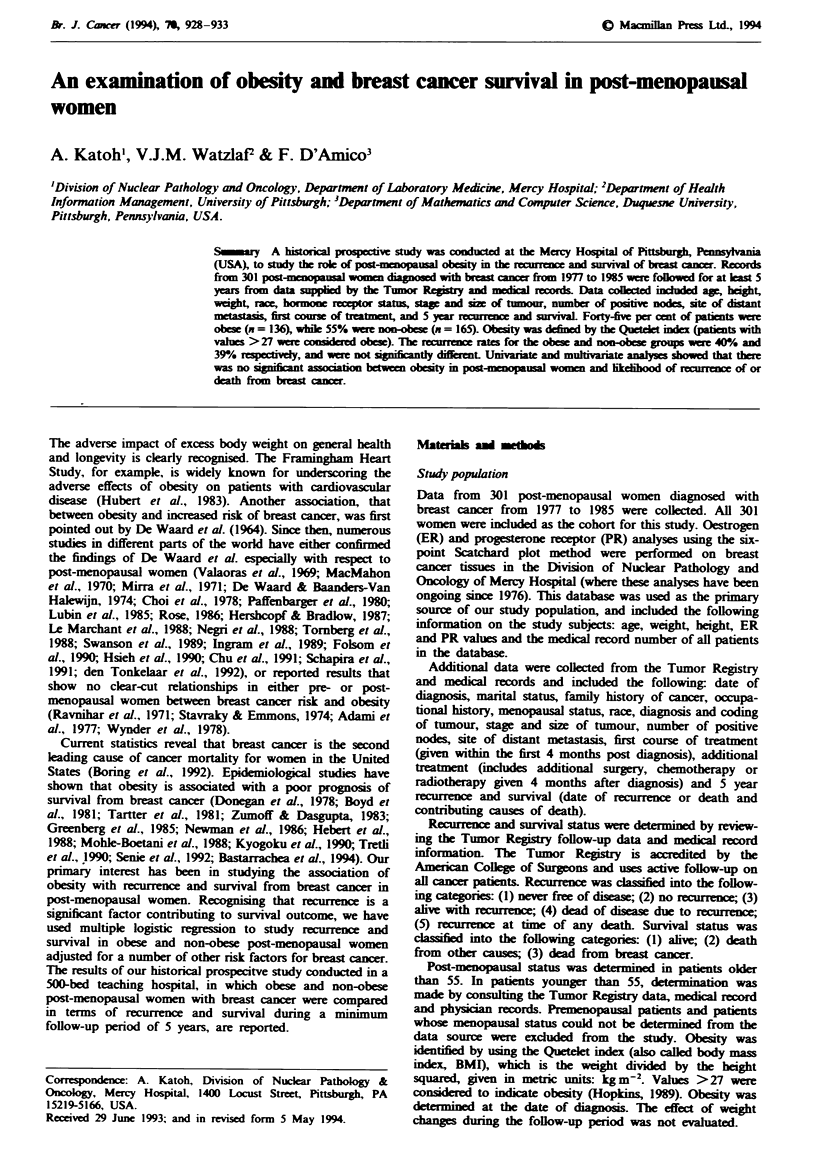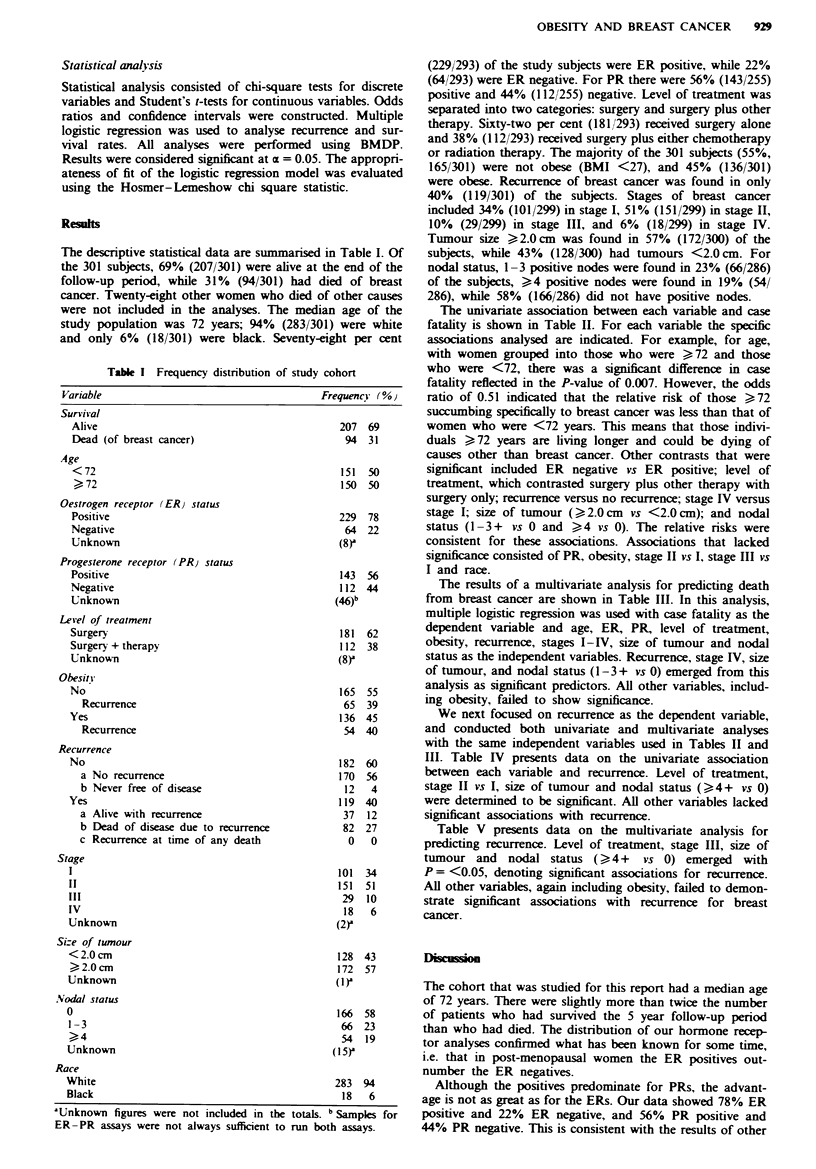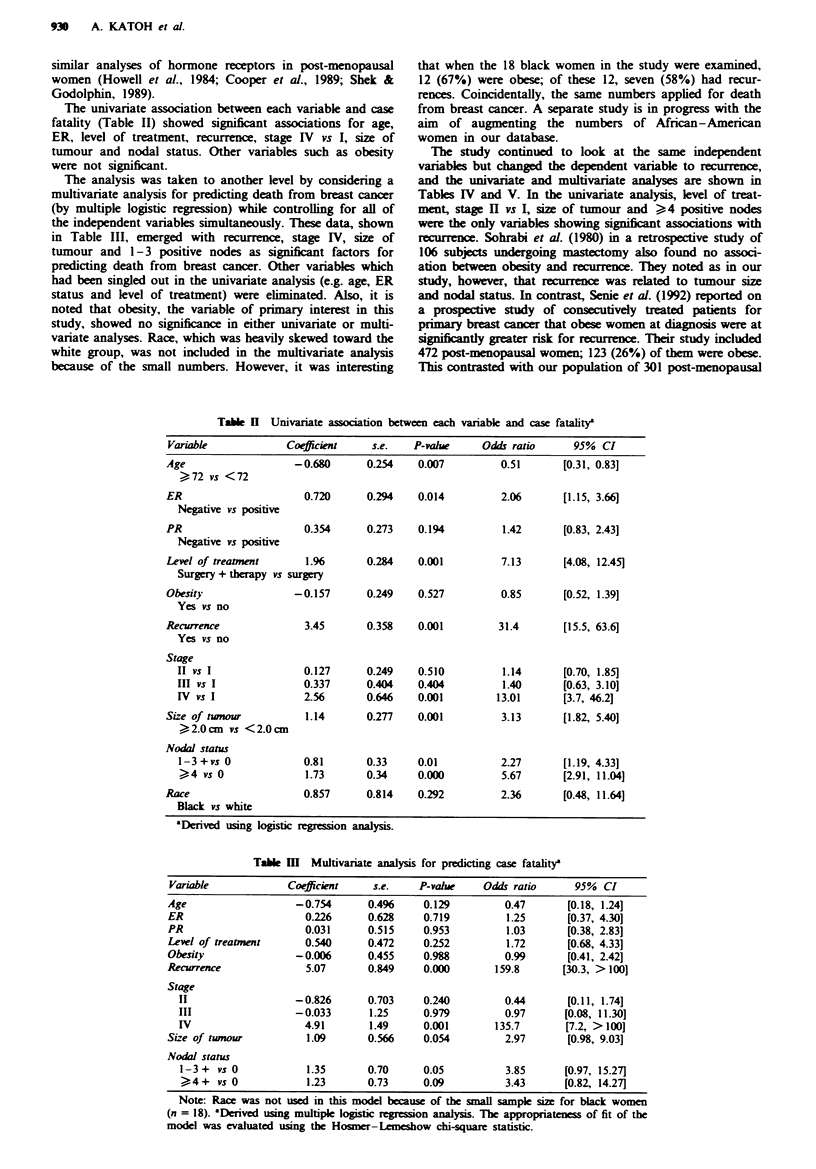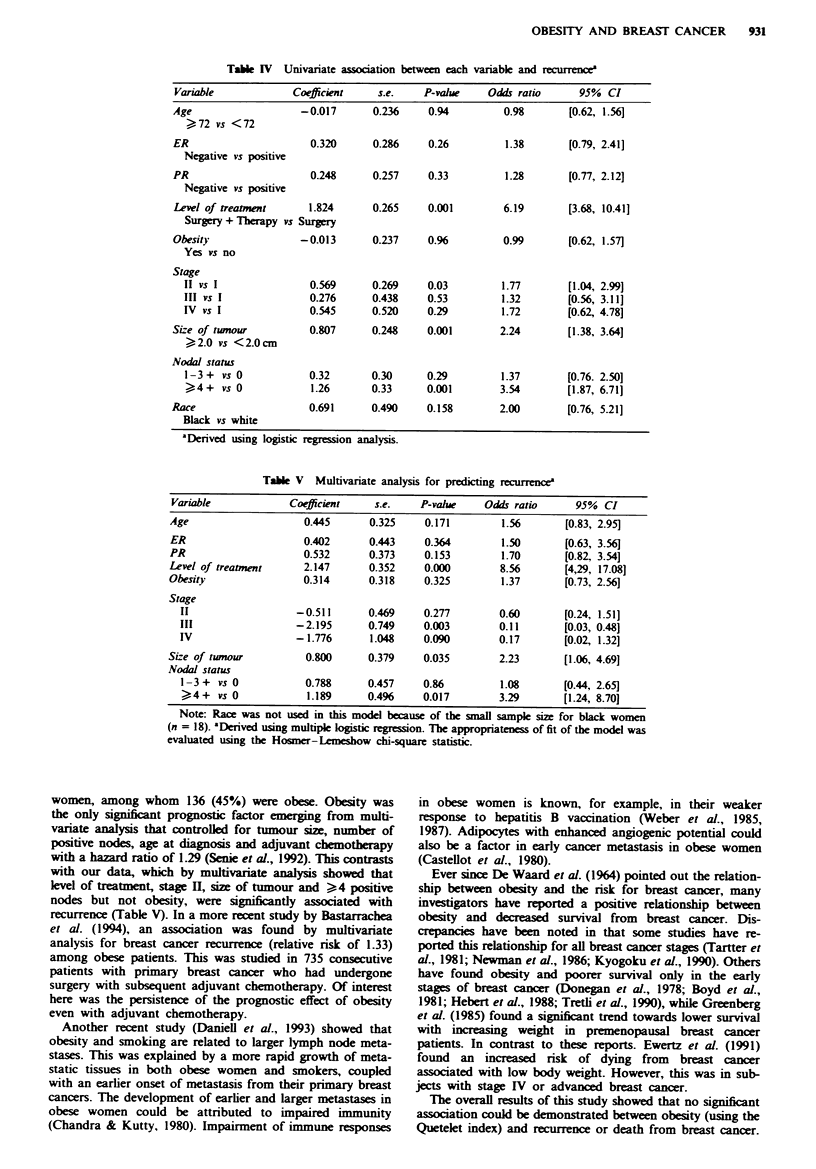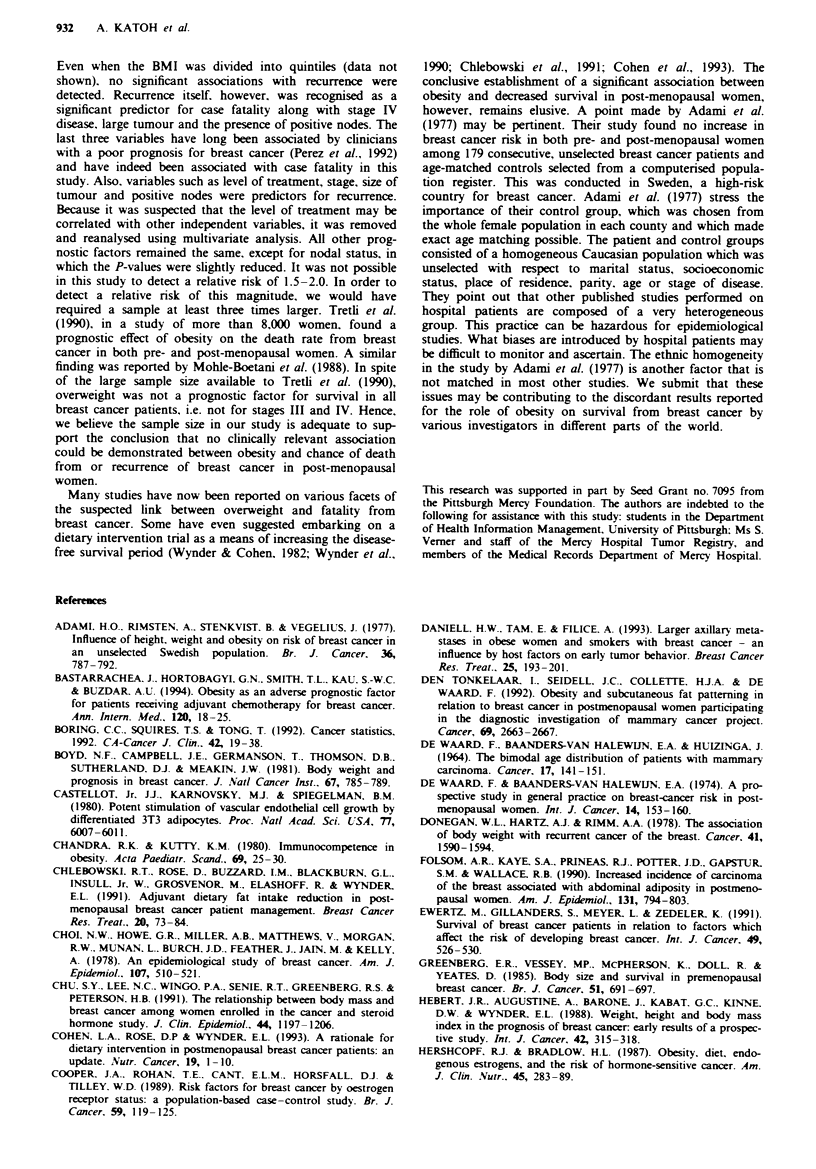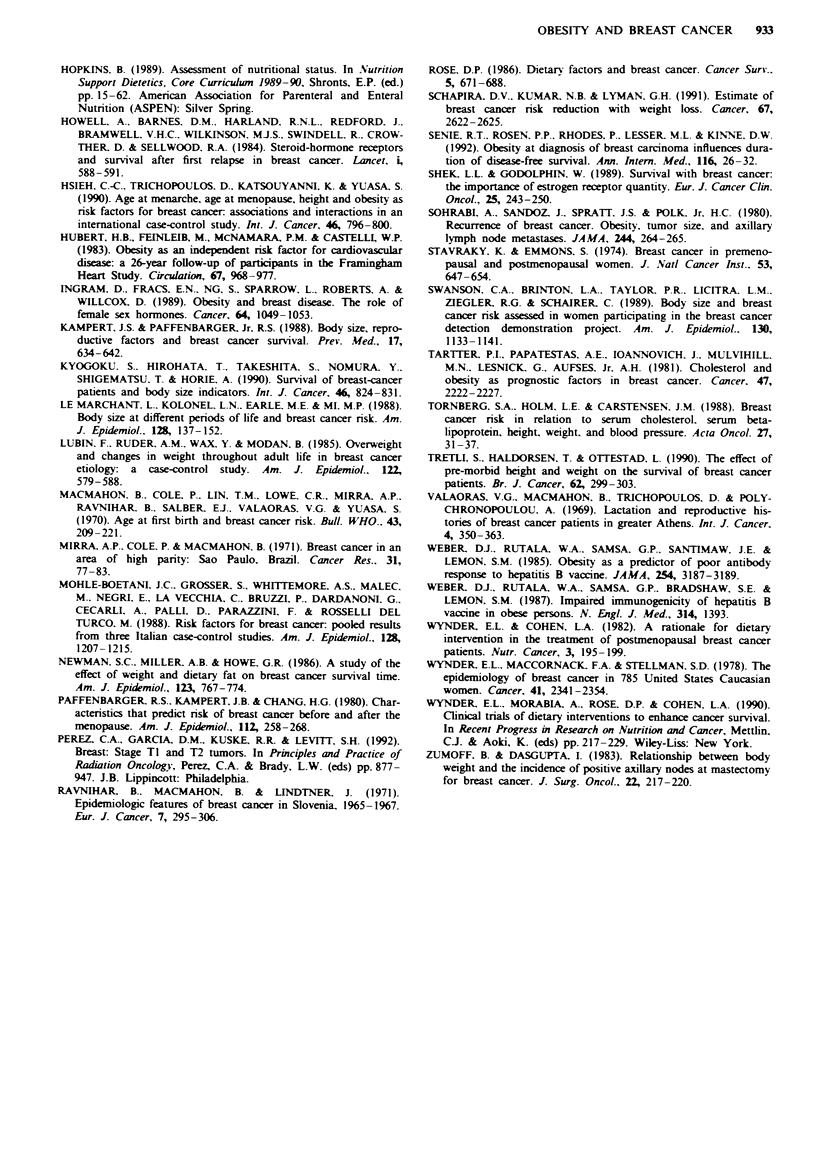# An examination of obesity and breast cancer survival in post-menopausal women.

**DOI:** 10.1038/bjc.1994.422

**Published:** 1994-11

**Authors:** A. Katoh, V. J. Watzlaf, F. D'Amico

**Affiliations:** Department of Laboratory Medicine, Mercy Hospital, Pittsburgh, Pennsylvania 15219-5166.

## Abstract

A historical prospective study was conducted at the Mercy Hospital of Pittsburgh, Pennsylvania (USA), to study the role of post-menopausal obesity in the recurrence and survival of breast cancer. Records from 301 post-menopausal women diagnosed with breast cancer from 1977 to 1985 were followed for at least 5 years from data supplied by the Tumor Registry and medical records. Data collected included age, height, weight, race, hormone receptor status, stage and size of tumour, number of positive nodes, site of distant metastasis, first course of treatment, and 5 year recurrence and survival. Forty-five per cent of patients were obese (n = 136), while 55% were non-obese (n = 165). Obesity was defined by the Quetelet index (patients with values > 27 were considered obese). The recurrence rates for the obese and non-obese groups were 40% and 39% respectively, and were not significantly different. Univariate and multivariate analyses showed that there was no significant association between obesity in post-menopausal women and likelihood of recurrence of or death from breast cancer.


Br. J. Cancer (1994), 70, 928-933 © Macmillan Press Ltd., 1994

### An examination of obesity and breast cancer survival in post-menopausal women

A. Katoh[1], V.J.M. Watzlaf[2] & F. D'Amico[3]

[1]Division of Nuclear Pathology and Oncology, Department of Laboratory Medicine, Mercy Hospital; [2]Department of Health Information Management, University of Pittsburgh; [3]Department of Mathematics and Computer Science, Duquesne University, Pittsburgh, Pennsylvania, USA.

**Summary** A historical prospective study was conducted at the Mercy Hospital of Pittsburgh, Pennsylvania (USA), to study the role of post-menopausal obesity in the recurrence and survival of breast cancer. Records from 301 post-menopausal women diagnosed with breast cancer from 1977 to 1985 were followed for at least 5 years from data supplied by the Tumor Registry and medical records. Data collected included age, height, weight, race, hormone receptor status, stage and size of tumour, number of positive nodes, site of distant metastasis, first course of treatment, and 5 year recurrence and survival. Forty-five per cent of patients were obese ($n = 136$), while 55% were non-obese ($n = 165$). Obesity was defined by the Quetelet index (patients with values >27 were considered obese). The recurrence rates for the obese and non-obese groups were 40% and 39% respectively, and were not significantly different. Univariate and multivariate analyses showed that there was no significant association between obesity in post-menopausal women and likelihood of recurrence of or death from breast cancer.

The adverse impact of excess body weight on general health and longevity is clearly recognised. The Framingham Heart Study, for example, is widely known for underscoring the adverse effects of obesity on patients with cardiovascular disease (Hubert *et al.*, 1983). Another association, that between obesity and increased risk of breast cancer, was first pointed out by De Waard *et al.* (1964). Since then, numerous studies in different parts of the world have either confirmed the findings of De Waard *et al.* especially with respect to post-menopausal women (Valaoras *et al.*, 1969; MacMahon *et al.*, 1970; Mirra *et al.*, 1971; De Waard & Baanders-Van Halewijn, 1974; Choi *et al.*, 1978; Paffenbarger *et al.*, 1980; Lubin *et al.*, 1985; Rose, 1986; Hershcopf & Bradlow, 1987; Le Marchant *et al.*, 1988; Negri *et al.*, 1988; Tornberg *et al.*, 1988; Swanson *et al.*, 1989; Ingram *et al.*, 1989; Folsom *et al.*, 1990; Hsieh *et al.*, 1990; Chu *et al.*, 1991; Schapira *et al.*, 1991; den Tonkelaar *et al.*, 1992), or reported results that show no clear-cut relationships in either pre- or post-menopausal women between breast cancer risk and obesity (Ravnihar *et al.*, 1971; Stavraky & Emmons, 1974; Adami *et al.*, 1977; Wynder *et al.*, 1978).

Current statistics reveal that breast cancer is the second leading cause of cancer mortality for women in the United States (Boring *et al.*, 1992). Epidemiological studies have shown that obesity is associated with a poor prognosis of survival from breast cancer (Donegan *et al.*, 1978; Boyd *et al.*, 1981; Tartter *et al.*, 1981; Zumoff & Dasgupta, 1983; Greenberg *et al.*, 1985; Newman *et al.*, 1986; Hebert *et al.*, 1988; Mohle-Boetani *et al.*, 1988; Kyogoku *et al.*, 1990; Tretli *et al.*, 1990; Senie *et al.*, 1992; Bastarrachea *et al.*, 1994). Our primary interest has been in studying the association of obesity with recurrence and survival from breast cancer in post-menopausal women. Recognising that recurrence is a significant factor contributing to survival outcome, we have used multiple logistic regression to study recurrence and survival in obese and non-obese post-menopausal women adjusted for a number of other risk factors for breast cancer. The results of our historical prospecitve study conducted in a 500-bed teaching hospital, in which obese and non-obese post-menopausal women with breast cancer were compared in terms of recurrence and survival during a minimum follow-up period of 5 years, are reported.

Correspondence: A. Katoh, Division of Nuclear Pathology & Oncology, Mercy Hospital, 1400 Locust Street, Pittsburgh, PA 15219-5166, USA.
Received 29 June 1993; and in revised form 5 May 1994.

### Materials and methods

### *Study population*

Data from 301 post-menopausal women diagnosed with breast cancer from 1977 to 1985 were collected. All 301 women were included as the cohort for this study. Oestrogen (ER) and progesterone receptor (PR) analyses using the six-point Scatchard plot method were performed on breast cancer tissues in the Division of Nuclear Pathology and Oncology of Mercy Hospital (where these analyses have been ongoing since 1976). This database was used as the primary source of our study population, and included the following information on the study subjects: age, weight, height, ER and PR values and the medical record number of all patients in the database.

Additional data were collected from the Tumor Registry and medical records and included the following: date of diagnosis, marital status, family history of cancer, occupational history, menopausal status, race, diagnosis and coding of tumour, stage and size of tumour, number of positive nodes, site of distant metastasis, first course of treatment (given within the first 4 months post diagnosis), additional treatment (includes additional surgery, chemotherapy or radiotherapy given 4 months after diagnosis) and 5 year recurrence and survival (date of recurrence or death and contributing causes of death).

Recurrence and survival status were determined by reviewing the Tumor Registry follow-up data and medical record information. The Tumor Registry is accredited by the American College of Surgeons and uses active follow-up on all cancer patients. Recurrence was classified into the following categories: (1) never free of disease; (2) no recurrence; (3) alive with recurrence; (4) dead of disease due to recurrence; (5) recurrence at time of any death. Survival status was classified into the following categories: (1) alive; (2) death from other causes; (3) dead from breast cancer.

Post-menopausal status was determined in patients older than 55. In patients younger than 55, determination was made by consulting the Tumor Registry data, medical record and physician records. Premenopausal patients and patients whose menopausal status could not be determined from the data source were excluded from the study. Obesity was identified by using the Quetelet index (also called body mass index, BMI), which is the weight divided by the height squared, given in metric units: $kg\,m^{-2}$. Values >27 were considered to indicate obesity (Hopkins, 1989). Obesity was determined at the date of diagnosis. The effect of weight changes during the follow-up period was not evaluated.

*Statistical analysis*

Statistical analysis consisted of chi-square tests for discrete variables and Student's *t*-tests for continuous variables. Odds ratios and confidence intervals were constructed. Multiple logistic regression was used to analyse recurrence and survival rates. All analyses were performed using BMDP. Results were considered significant at $\alpha = 0.05$. The appropriateness of fit of the logistic regression model was evaluated using the Hosmer–Lemeshow chi square statistic.

### Results

The descriptive statistical data are summarised in Table I. Of the 301 subjects, 69% (207/301) were alive at the end of the follow-up period, while 31% (94/301) had died of breast cancer. Twenty-eight other women who died of other causes were not included in the analyses. The median age of the study population was 72 years; 94% (283/301) were white and only 6% (18/301) were black. Seventy-eight per cent (229/293) of the study subjects were ER positive, while 22% (64/293) were ER negative. For PR there were 56% (143/255) positive and 44% (112/255) negative. Level of treatment was separated into two categories: surgery and surgery plus other therapy. Sixty-two per cent (181/293) received surgery alone and 38% (112/293) received surgery plus either chemotherapy or radiation therapy. The majority of the 301 subjects (55%, 165/301) were not obese (BMI <27), and 45% (136/301) were obese. Recurrence of breast cancer was found in only 40% (119/301) of the subjects. Stages of breast cancer included 34% (101/299) in stage I, 51% (151/299) in stage II, 10% (29/299) in stage III, and 6% (18/299) in stage IV. Tumour size ≥2.0 cm was found in 57% (172/300) of the subjects, while 43% (128/300) had tumours <2.0 cm. For nodal status, 1–3 positive nodes were found in 23% (66/286) of the subjects, ≥4 positive nodes were found in 19% (54/286), while 58% (166/286) did not have positive nodes.

**Table I** Frequency distribution of study cohort

| *Variable* | *Frequency* | *(%)* |
|---|---|---|
| *Survival* | | |
| Alive | 207 | 69 |
| Dead (of breast cancer) | 94 | 31 |
| *Age* | | |
| <72 | 151 | 50 |
| ≥72 | 150 | 50 |
| *Oestrogen receptor (ER) status* | | |
| Positive | 229 | 78 |
| Negative | 64 | 22 |
| Unknown | (8)[a] | |
| *Progesterone receptor (PR) status* | | |
| Positive | 143 | 56 |
| Negative | 112 | 44 |
| Unknown | (46)[b] | |
| *Level of treatment* | | |
| Surgery | 181 | 62 |
| Surgery + therapy | 112 | 38 |
| Unknown | (8)[a] | |
| *Obesity* | | |
| No | 165 | 55 |
| Recurrence | 65 | 39 |
| Yes | 136 | 45 |
| Recurrence | 54 | 40 |
| *Recurrence* | | |
| No | 182 | 60 |
| a No recurrence | 170 | 56 |
| b Never free of disease | 12 | 4 |
| Yes | 119 | 40 |
| a Alive with recurrence | 37 | 12 |
| b Dead of disease due to recurrence | 82 | 27 |
| c Recurrence at time of any death | 0 | 0 |
| *Stage* | | |
| I | 101 | 34 |
| II | 151 | 51 |
| III | 29 | 10 |
| IV | 18 | 6 |
| Unknown | (2)[a] | |
| *Size of tumour* | | |
| <2.0 cm | 128 | 43 |
| ≥2.0 cm | 172 | 57 |
| Unknown | (1)[a] | |
| *Nodal status* | | |
| 0 | 166 | 58 |
| 1–3 | 66 | 23 |
| ≥4 | 54 | 19 |
| Unknown | (15)[a] | |
| *Race* | | |
| White | 283 | 94 |
| Black | 18 | 6 |

[a]Unknown figures were not included in the totals. [b]Samples for ER–PR assays were not always sufficient to run both assays.

The univariate association between each variable and case fatality is shown in Table II. For each variable the specific associations analysed are indicated. For example, for age, with women grouped into those who were ≥72 and those who were <72, there was a significant difference in case fatality reflected in the *P*-value of 0.007. However, the odds ratio of 0.51 indicated that the relative risk of those ≥72 succumbing specifically to breast cancer was less than that of women who were <72 years. This means that those individuals ≥72 years are living longer and could be dying of causes other than breast cancer. Other contrasts that were significant included ER negative *vs* ER positive; level of treatment, which contrasted surgery plus other therapy with surgery only; recurrence versus no recurrence; stage IV versus stage I; size of tumour (≥2.0 cm *vs* <2.0 cm); and nodal status (1–3+ *vs* 0 and ≥4 *vs* 0). The relative risks were consistent for these associations. Associations that lacked significance consisted of PR, obesity, stage II *vs* I, stage III *vs* I and race.

The results of a multivariate analysis for predicting death from breast cancer are shown in Table III. In this analysis, multiple logistic regression was used with case fatality as the dependent variable and age, ER, PR, level of treatment, obesity, recurrence, stages I–IV, size of tumour and nodal status as the independent variables. Recurrence, stage IV, size of tumour, and nodal status (1–3+ *vs* 0) emerged from this analysis as significant predictors. All other variables, including obesity, failed to show significance.

We next focused on recurrence as the dependent variable, and conducted both univariate and multivariate analyses with the same independent variables used in Tables II and III. Table IV presents data on the univariate association between each variable and recurrence. Level of treatment, stage II *vs* I, size of tumour and nodal status (≥4+ *vs* 0) were determined to be significant. All other variables lacked significant associations with recurrence.

Table V presents data on the multivariate analysis for predicting recurrence. Level of treatment, stage III, size of tumour and nodal status (≥4+ *vs* 0) emerged with $P = <0.05$, denoting significant associations for recurrence. All other variables, again including obesity, failed to demonstrate significant associations with recurrence for breast cancer.

### Discussion

The cohort that was studied for this report had a median age of 72 years. There were slightly more than twice the number of patients who had survived the 5 year follow-up period than who had died. The distribution of our hormone receptor analyses confirmed what has been known for some time, i.e. that in post-menopausal women the ER positives outnumber the ER negatives.

Although the positives predominate for PRs, the advantage is not as great as for the ERs. Our data showed 78% ER positive and 22% ER negative, and 56% PR positive and 44% PR negative. This is consistent with the results of other similar analyses of hormone receptors in post-menopausal women (Howell et al., 1984; Cooper et al., 1989; Shek & Godolphin, 1989).

The univariate association between each variable and case fatality (Table II) showed significant associations for age, ER, level of treatment, recurrence, stage IV vs I, size of tumour and nodal status. Other variables such as obesity were not significant.

The analysis was taken to another level by considering a multivariate analysis for predicting death from breast cancer (by multiple logistic regression) while controlling for all of the independent variables simultaneously. These data, shown in Table III, emerged with recurrence, stage IV, size of tumour and 1–3 positive nodes as significant factors for predicting death from breast cancer. Other variables which had been singled out in the univariate analysis (e.g. age, ER status and level of treatment) were eliminated. Also, it is noted that obesity, the variable of primary interest in this study, showed no significance in either univariate or multivariate analyses. Race, which was heavily skewed toward the white group, was not included in the multivariate analysis because of the small numbers. However, it was interesting that when the 18 black women in the study were examined, 12 (67%) were obese; of these 12, seven (58%) had recurrences. Coincidentally, the same numbers applied for death from breast cancer. A separate study is in progress with the aim of augmenting the numbers of African-American women in our database.

The study continued to look at the same independent variables but changed the dependent variable to recurrence, and the univariate and multivariate analyses are shown in Tables IV and V. In the univariate analysis, level of treatment, stage II vs I, size of tumour and ⩾4 positive nodes were the only variables showing significant associations with recurrence. Sohrabi et al. (1980) in a retrospective study of 106 subjects undergoing mastectomy also found no association between obesity and recurrence. They noted as in our study, however, that recurrence was related to tumour size and nodal status. In contrast, Senie et al. (1992) reported on a prospective study of consecutively treated patients for primary breast cancer that obese women at diagnosis were at significantly greater risk for recurrence. Their study included 472 post-menopausal women; 123 (26%) of them were obese. This contrasted with our population of 301 post-menopausal

**Table II** Univariate association between each variable and case fatality[a]

| Variable | Coefficient | s.e. | P-value | Odds ratio | 95% CI |
|---|---|---|---|---|---|
| *Age* ⩾72 vs <72 | −0.680 | 0.254 | 0.007 | 0.51 | [0.31, 0.83] |
| *ER* Negative vs positive | 0.720 | 0.294 | 0.014 | 2.06 | [1.15, 3.66] |
| *PR* Negative vs positive | 0.354 | 0.273 | 0.194 | 1.42 | [0.83, 2.43] |
| *Level of treatment* Surgery + therapy vs surgery | 1.96 | 0.284 | 0.001 | 7.13 | [4.08, 12.45] |
| *Obesity* Yes vs no | −0.157 | 0.249 | 0.527 | 0.85 | [0.52, 1.39] |
| *Recurrence* Yes vs no | 3.45 | 0.358 | 0.001 | 31.4 | [15.5, 63.6] |
| *Stage* | | | | | |
| II vs I | 0.127 | 0.249 | 0.510 | 1.14 | [0.70, 1.85] |
| III vs I | 0.337 | 0.404 | 0.404 | 1.40 | [0.63, 3.10] |
| IV vs I | 2.56 | 0.646 | 0.001 | 13.01 | [3.7, 46.2] |
| *Size of tumour* ⩾2.0 cm vs <2.0 cm | 1.14 | 0.277 | 0.001 | 3.13 | [1.82, 5.40] |
| *Nodal status* | | | | | |
| 1–3 + vs 0 | 0.81 | 0.33 | 0.01 | 2.27 | [1.19, 4.33] |
| ⩾4 vs 0 | 1.73 | 0.34 | 0.000 | 5.67 | [2.91, 11.04] |
| *Race* Black vs white | 0.857 | 0.814 | 0.292 | 2.36 | [0.48, 11.64] |

[a]Derived using logistic regression analysis.

**Table III** Multivariate analysis for predicting case fatality[a]

| Variable | Coefficient | s.e. | P-value | Odds ratio | 95% CI |
|---|---|---|---|---|---|
| *Age* | −0.754 | 0.496 | 0.129 | 0.47 | [0.18, 1.24] |
| *ER* | 0.226 | 0.628 | 0.719 | 1.25 | [0.37, 4.30] |
| *PR* | 0.031 | 0.515 | 0.953 | 1.03 | [0.38, 2.83] |
| *Level of treatment* | 0.540 | 0.472 | 0.252 | 1.72 | [0.68, 4.33] |
| *Obesity* | −0.006 | 0.455 | 0.988 | 0.99 | [0.41, 2.42] |
| *Recurrence* | 5.07 | 0.849 | 0.000 | 159.8 | [30.3, >100] |
| *Stage* | | | | | |
| II | −0.826 | 0.703 | 0.240 | 0.44 | [0.11, 1.74] |
| III | −0.033 | 1.25 | 0.979 | 0.97 | [0.08, 11.30] |
| IV | 4.91 | 1.49 | 0.001 | 135.7 | [7.2, >100] |
| *Size of tumour* | 1.09 | 0.566 | 0.054 | 2.97 | [0.98, 9.03] |
| *Nodal status* | | | | | |
| 1–3 + vs 0 | 1.35 | 0.70 | 0.05 | 3.85 | [0.97, 15.27] |
| ⩾4 + vs 0 | 1.23 | 0.73 | 0.09 | 3.43 | [0.82, 14.27] |

Note: Race was not used in this model because of the small sample size for black women ($n$ = 18). [a]Derived using multiple logistic regression analysis. The appropriateness of fit of the model was evaluated using the Hosmer–Lemeshow chi-square statistic.

**Table IV** Univariate association between each variable and recurrence[a]

| *Variable* | *Coefficient* | *s.e.* | *P-value* | *Odds ratio* | *95% CI* |
|---|---|---|---|---|---|
| *Age* | −0.017 | 0.236 | 0.94 | 0.98 | [0.62, 1.56] |
| ≥72 *vs* <72 | | | | | |
| *ER* | 0.320 | 0.286 | 0.26 | 1.38 | [0.79, 2.41] |
| Negative *vs* positive | | | | | |
| *PR* | 0.248 | 0.257 | 0.33 | 1.28 | [0.77, 2.12] |
| Negative *vs* positive | | | | | |
| *Level of treatment* | 1.824 | 0.265 | 0.001 | 6.19 | [3.68, 10.41] |
| Surgery + Therapy *vs* Surgery | | | | | |
| *Obesity* | −0.013 | 0.237 | 0.96 | 0.99 | [0.62, 1.57] |
| Yes *vs* no | | | | | |
| *Stage* | | | | | |
| II *vs* I | 0.569 | 0.269 | 0.03 | 1.77 | [1.04, 2.99] |
| III *vs* I | 0.276 | 0.438 | 0.53 | 1.32 | [0.56, 3.11] |
| IV *vs* I | 0.545 | 0.520 | 0.29 | 1.72 | [0.62, 4.78] |
| *Size of tumour* | 0.807 | 0.248 | 0.001 | 2.24 | [1.38, 3.64] |
| ≥2.0 *vs* <2.0 cm | | | | | |
| *Nodal status* | | | | | |
| 1–3+ *vs* 0 | 0.32 | 0.30 | 0.29 | 1.37 | [0.76. 2.50] |
| ≥4+ *vs* 0 | 1.26 | 0.33 | 0.001 | 3.54 | [1.87, 6.71] |
| *Race* | 0.691 | 0.490 | 0.158 | 2.00 | [0.76, 5.21] |
| Black *vs* white | | | | | |

[a]Derived using logistic regression analysis.

**Table V** Multivariate analysis for predicting recurrence[a]

| *Variable* | *Coefficient* | *s.e.* | *P-value* | *Odds ratio* | *95% CI* |
|---|---|---|---|---|---|
| *Age* | 0.445 | 0.325 | 0.171 | 1.56 | [0.83, 2.95] |
| *ER* | 0.402 | 0.443 | 0.364 | 1.50 | [0.63, 3.56] |
| *PR* | 0.532 | 0.373 | 0.153 | 1.70 | [0.82, 3.54] |
| *Level of treatment* | 2.147 | 0.352 | 0.000 | 8.56 | [4,29, 17.08] |
| *Obesity* | 0.314 | 0.318 | 0.325 | 1.37 | [0.73, 2.56] |
| *Stage* | | | | | |
| II | −0.511 | 0.469 | 0.277 | 0.60 | [0.24, 1.51] |
| III | −2.195 | 0.749 | 0.003 | 0.11 | [0.03, 0.48] |
| IV | −1.776 | 1.048 | 0.090 | 0.17 | [0.02, 1.32] |
| *Size of tumour* | 0.800 | 0.379 | 0.035 | 2.23 | [1.06, 4.69] |
| *Nodal status* | | | | | |
| 1–3+ *vs* 0 | 0.788 | 0.457 | 0.86 | 1.08 | [0.44, 2.65] |
| ≥4+ *vs* 0 | 1.189 | 0.496 | 0.017 | 3.29 | [1.24, 8.70] |

Note: Race was not used in this model because of the small sample size for black women ($n$ = 18). [a]Derived using multiple logistic regression. The appropriateness of fit of the model was evaluated using the Hosmer–Lemeshow chi-square statistic.

women, among whom 136 (45%) were obese. Obesity was the only significant prognostic factor emerging from multivariate analysis that controlled for tumour size, number of positive nodes, age at diagnosis and adjuvant chemotherapy with a hazard ratio of 1.29 (Senie *et al.*, 1992). This contrasts with our data, which by multivariate analysis showed that level of treatment, stage II, size of tumour and ≥4 positive nodes but not obesity, were significantly associated with recurrence (Table V). In a more recent study by Bastarrachea *et al.* (1994), an association was found by multivariate analysis for breast cancer recurrence (relative risk of 1.33) among obese patients. This was studied in 735 consecutive patients with primary breast cancer who had undergone surgery with subsequent adjuvant chemotherapy. Of interest here was the persistence of the prognostic effect of obesity even with adjuvant chemotherapy.

Another recent study (Daniell *et al.*, 1993) showed that obesity and smoking are related to larger lymph node metastases. This was explained by a more rapid growth of metastatic tissues in both obese women and smokers, coupled with an earlier onset of metastasis from their primary breast cancers. The development of earlier and larger metastases in obese women could be attributed to impaired immunity (Chandra & Kutty, 1980). Impairment of immune responses in obese women is known, for example, in their weaker response to hepatitis B vaccination (Weber *et al.*, 1985, 1987). Adipocytes with enhanced angiogenic potential could also be a factor in early cancer metastasis in obese women (Castellot *et al.*, 1980).

Ever since De Waard *et al.* (1964) pointed out the relationship between obesity and the risk for breast cancer, many investigators have reported a positive relationship between obesity and decreased survival from breast cancer. Discrepancies have been noted in that some studies have reported this relationship for all breast cancer stages (Tartter *et al.*, 1981; Newman *et al.*, 1986; Kyogoku *et al.*, 1990). Others have found obesity and poorer survival only in the early stages of breast cancer (Donegan *et al.*, 1978; Boyd *et al.*, 1981; Hebert *et al.*, 1988; Tretli *et al.*, 1990), while Greenberg *et al.* (1985) found a significant trend towards lower survival with increasing weight in premenopausal breast cancer patients. In contrast to these reports. Ewertz *et al.* (1991) found an increased risk of dying from breast cancer associated with low body weight. However, this was in subjects with stage IV or advanced breast cancer.

The overall results of this study showed that no significant association could be demonstrated between obesity (using the Quetelet index) and recurrence or death from breast cancer.

Even when the BMI was divided into quintiles (data not shown), no significant associations with recurrence were detected. Recurrence itself, however, was recognised as a significant predictor for case fatality along with stage IV disease, large tumour and the presence of positive nodes. The last three variables have long been associated by clinicians with a poor prognosis for breast cancer (Perez et al., 1992) and have indeed been associated with case fatality in this study. Also, variables such as level of treatment, stage, size of tumour and positive nodes were predictors for recurrence. Because it was suspected that the level of treatment may be correlated with other independent variables, it was removed and reanalysed using multivariate analysis. All other prognostic factors remained the same, except for nodal status, in which the P-values were slightly reduced. It was not possible in this study to detect a relative risk of 1.5–2.0. In order to detect a relative risk of this magnitude, we would have required a sample at least three times larger. Tretli et al. (1990), in a study of more than 8,000 women, found a prognostic effect of obesity on the death rate from breast cancer in both pre- and post-menopausal women. A similar finding was reported by Mohle-Boetani et al. (1988). In spite of the large sample size available to Tretli et al. (1990), overweight was not a prognostic factor for survival in all breast cancer patients, i.e. not for stages III and IV. Hence, we believe the sample size in our study is adequate to support the conclusion that no clinically relevant association could be demonstrated between obesity and chance of death from or recurrence of breast cancer in post-menopausal women.

Many studies have now been reported on various facets of the suspected link between overweight and fatality from breast cancer. Some have even suggested embarking on a dietary intervention trial as a means of increasing the disease-free survival period (Wynder & Cohen, 1982; Wynder et al., 1990; Chlebowski et al., 1991; Cohen et al., 1993). The conclusive establishment of a significant association between obesity and decreased survival in post-menopausal women, however, remains elusive. A point made by Adami et al. (1977) may be pertinent. Their study found no increase in breast cancer risk in both pre- and post-menopausal women among 179 consecutive, unselected breast cancer patients and age-matched controls selected from a computerised population register. This was conducted in Sweden, a high-risk country for breast cancer. Adami et al. (1977) stress the importance of their control group, which was chosen from the whole female population in each county and which made exact age matching possible. The patient and control groups consisted of a homogeneous Caucasian population which was unselected with respect to marital status, socioeconomic status, place of residence, parity, age or stage of disease. They point out that other published studies performed on hospital patients are composed of a very heterogeneous group. This practice can be hazardous for epidemiological studies. What biases are introduced by hospital patients may be difficult to monitor and ascertain. The ethnic homogeneity in the study by Adami et al. (1977) is another factor that is not matched in most other studies. We submit that these issues may be contributing to the discordant results reported for the role of obesity on survival from breast cancer by various investigators in different parts of the world.

This research was supported in part by Seed Grant no. 7095 from the Pittsburgh Mercy Foundation. The authors are indebted to the following for assistance with this study: students in the Department of Health Information Management, University of Pittsburgh; Ms S. Verner and staff of the Mercy Hospital Tumor Registry, and members of the Medical Records Department of Mercy Hospital.

### References

ADAMI, H.O., RIMSTEN, A., STENKVIST, B. & VEGELIUS, J. (1977). Influence of height, weight and obesity on risk of breast cancer in an unselected Swedish population. *Br. J. Cancer*, **36**, 787–792.

BASTARRACHEA, J., HORTOBAGYI, G.N., SMITH, T.L., KAU, S.-W.C. & BUZDAR, A.U. (1994). Obesity as an adverse prognostic factor for patients receiving adjuvant chemotherapy for breast cancer. *Ann. Intern. Med.*, **120**, 18–25.

BORING, C.C., SQUIRES, T.S. & TONG, T. (1992). Cancer statistics, 1992. *CA-Cancer J. Clin.*, **42**, 19–38.

BOYD, N.F., CAMPBELL, J.E., GERMANSON, T., THOMSON, D.B., SUTHERLAND, D.J. & MEAKIN, J.W. (1981). Body weight and prognosis in breast cancer. *J. Natl Cancer Inst.*, **67**, 785–789.

CASTELLOT, Jr, J.J., KARNOVSKY, M.J. & SPIEGELMAN, B.M. (1980). Potent stimulation of vascular endothelial cell growth by differentiated 3T3 adipocytes. *Proc. Natl Acad. Sci. USA*, **77**, 6007–6011.

CHANDRA, R.K. & KUTTY, K.M. (1980). Immunocompetence in obesity. *Acta Paediatr. Scand.*, **69**, 25–30.

CHLEBOWSKI, R.T., ROSE, D., BUZZARD, I.M., BLACKBURN, G.L., INSULL, Jr, W., GROSVENOR, M., ELASHOFF, R. & WYNDER, E.L. (1991). Adjuvant dietary fat intake reduction in post-menopausal breast cancer patient management. *Breast Cancer Res. Treat.*, **20**, 73–84.

CHOI, N.W., HOWE, G.R., MILLER, A.B., MATTHEWS, V., MORGAN, R.W., MUNAN, L., BURCH, J.D., FEATHER, J., JAIN, M. & KELLY, A. (1978). An epidemiological study of breast cancer. *Am. J. Epidemiol.*, **107**, 510–521.

CHU, S.Y., LEE, N.C., WINGO, P.A., SENIE, R.T., GREENBERG, R.S. & PETERSON, H.B. (1991). The relationship between body mass and breast cancer among women enrolled in the cancer and steroid hormone study. *J. Clin. Epidemiol.*, **44**, 1197–1206.

COHEN, L.A., ROSE, D.P & WYNDER, E.L. (1993). A rationale for dietary intervention in postmenopausal breast cancer patients: an update. *Nutr. Cancer*, **19**, 1–10.

COOPER, J.A., ROHAN, T.E., CANT, E.L.M., HORSFALL, D.J. & TILLEY, W.D. (1989). Risk factors for breast cancer by oestrogen receptor status: a population-based case–control study. *Br. J. Cancer*, **59**, 119–125.

DANIELL, H.W., TAM, E. & FILICE, A. (1993). Larger axillary metastases in obese women and smokers with breast cancer – an influence by host factors on early tumor behavior. *Breast Cancer Res. Treat.*, **25**, 193–201.

DEN TONKELAAR, I., SEIDELL, J.C., COLLETTE, H.J.A. & DE WAARD, F. (1992). Obesity and subcutaneous fat patterning in relation to breast cancer in postmenopausal women participating in the diagnostic investigation of mammary cancer project. *Cancer*, **69**, 2663–2667.

DE WAARD, F., BAANDERS-VAN HALEWIJN, E.A. & HUIZINGA, J. (1964). The bimodal age distribution of patients with mammary carcinoma. *Cancer*, **17**, 141–151.

DE WAARD, F. & BAANDERS-VAN HALEWIJN, E.A. (1974). A prospective study in general practice on breast-cancer risk in post-menopausal women. *Int. J. Cancer*, **14**, 153–160.

DONEGAN, W.L., HARTZ, A.J. & RIMM, A.A. (1978). The association of body weight with recurrent cancer of the breast. *Cancer*, **41**, 1590–1594.

FOLSOM, A.R., KAYE, S.A., PRINEAS, R.J., POTTER, J.D., GAPSTUR, S.M. & WALLACE, R.B. (1990). Increased incidence of carcinoma of the breast associated with abdominal adiposity in postmenopausal women. *Am. J. Epidemiol.*, **131**, 794–803.

EWERTZ, M., GILLANDERS, S., MEYER, L. & ZEDELER, K. (1991). Survival of breast cancer patients in relation to factors which affect the risk of developing breast cancer. *Int. J. Cancer*, **49**, 526–530.

GREENBERG, E.R., VESSEY, MP., McPHERSON, K., DOLL, R. & YEATES, D. (1985). Body size and survival in premenopausal breast cancer. *Br. J. Cancer*, **51**, 691–697.

HEBERT, J.R., AUGUSTINE, A., BARONE, J., KABAT, G.C., KINNE, D.W. & WYNDER, E.L. (1988). Weight, height and body mass index in the prognosis of breast cancer: early results of a prospective study. *Int. J. Cancer*, **42**, 315–318.

HERSHCOPF, R.J. & BRADLOW, H.L. (1987). Obesity, diet, endogenous estrogens, and the risk of hormone-sensitive cancer. *Am. J. Clin. Nutr.*, **45**, 283–89.

HOPKINS, B. (1989). Assessment of nutritional status. In *Nutrition Support Dietetics, Core Curriculum 1989-90*, Shronts, E.P. (ed.) pp. 15-62. American Association for Parenteral and Enteral Nutrition (ASPEN): Silver Spring.

HOWELL, A., BARNES, D.M., HARLAND, R.N.L., REDFORD, J., BRAMWELL, V.H.C., WILKINSON, M.J.S., SWINDELL, R., CROWTHER, D. & SELLWOOD, R.A. (1984). Steroid-hormone receptors and survival after first relapse in breast cancer. *Lancet*, **i**, 588-591.

HSIEH, C.-C., TRICHOPOULOS, D., KATSOUYANNI, K. & YUASA, S. (1990). Age at menarche, age at menopause, height and obesity as risk factors for breast cancer: associations and interactions in an international case-control study. *Int. J. Cancer*, **46**, 796-800.

HUBERT, H.B., FEINLEIB, M., MCNAMARA, P.M. & CASTELLI, W.P. (1983). Obesity as an independent risk factor for cardiovascular disease: a 26-year follow-up of participants in the Framingham Heart Study. *Circulation*, **67**, 968-977.

INGRAM, D., FRACS, E.N., NG, S., SPARROW, L., ROBERTS, A. & WILLCOX, D. (1989). Obesity and breast disease. The role of female sex hormones. *Cancer*, **64**, 1049-1053.

KAMPERT, J.S. & PAFFENBARGER, Jr, R.S. (1988). Body size, reproductive factors and breast cancer survival. *Prev. Med.*, **17**, 634-642.

KYOGOKU, S., HIROHATA, T., TAKESHITA, S., NOMURA, Y., SHIGEMATSU, T. & HORIE, A. (1990). Survival of breast-cancer patients and body size indicators. *Int. J. Cancer*, **46**, 824-831.

LE MARCHANT, L., KOLONEL, L.N., EARLE, M.E. & MI, M.P. (1988). Body size at different periods of life and breast cancer risk. *Am. J. Epidemiol.*, **128**, 137-152.

LUBIN, F., RUDER, A.M., WAX, Y. & MODAN, B. (1985). Overweight and changes in weight throughout adult life in breast cancer etiology: a case-control study. *Am. J. Epidemiol.*, **122**, 579-588.

MACMAHON, B., COLE, P., LIN, T.M., LOWE, C.R., MIRRA, A.P., RAVNIHAR, B., SALBER, E.J., VALAORAS, V.G. & YUASA, S. (1970). Age at first birth and breast cancer risk. *Bull. WHO.*, **43**, 209-221.

MIRRA, A.P., COLE, P. & MACMAHON, B. (1971). Breast cancer in an area of high parity: Sao Paulo, Brazil. *Cancer Res.*, **31**, 77-83.

MOHLE-BOETANI, J.C., GROSSER, S., WHITTEMORE, A.S., MALEC, M., NEGRI, E., LA VECCHIA, C., BRUZZI, P., DARDANONI, G., CECARLI, A., PALLI, D., PARAZZINI, F. & ROSSELLI DEL TURCO, M. (1988). Risk factors for breast cancer: pooled results from three Italian case-control studies. *Am. J. Epidemiol.*, **128**, 1207-1215.

NEWMAN, S.C., MILLER, A.B. & HOWE, G.R. (1986). A study of the effect of weight and dietary fat on breast cancer survival time. *Am. J. Epidemiol.*, **123**, 767-774.

PAFFENBARGER, R.S., KAMPERT, J.B. & CHANG, H.G. (1980). Characteristics that predict risk of breast cancer before and after the menopause. *Am. J. Epidemiol.*, **112**, 258-268.

PEREZ, C.A., GARCIA, D.M., KUSKE, R.R. & LEVITT, S.H. (1992). Breast: Stage T1 and T2 tumors. In *Principles and Practice of Radiation Oncology*, Perez, C.A. & Brady, L.W. (eds) pp. 877-947. J.B. Lippincott: Philadelphia.

RAVNIHAR, B., MACMAHON, B. & LINDTNER, J. (1971). Epidemiologic features of breast cancer in Slovenia, 1965-1967. *Eur. J. Cancer*, **7**, 295-306.

ROSE, D.P. (1986). Dietary factors and breast cancer. *Cancer Surv.*, **5**, 671-688.

SCHAPIRA, D.V., KUMAR, N.B. & LYMAN, G.H. (1991). Estimate of breast cancer risk reduction with weight loss. *Cancer*, **67**, 2622-2625.

SENIE, R.T., ROSEN, P.P., RHODES, P., LESSER, M.L. & KINNE, D.W. (1992). Obesity at diagnosis of breast carcinoma influences duration of disease-free survival. *Ann. Intern. Med.*, **116**, 26-32.

SHEK, L.L. & GODOLPHIN, W. (1989). Survival with breast cancer: the importance of estrogen receptor quantity. *Eur. J. Cancer Clin. Oncol.*, **25**, 243-250.

SOHRABI, A., SANDOZ, J., SPRATT, J.S. & POLK, Jr, H.C. (1980). Recurrence of breast cancer. Obesity, tumor size, and axillary lymph node metastases. *JAMA*, **244**, 264-265.

STAVRAKY, K. & EMMONS, S. (1974). Breast cancer in premenopausal and postmenopausal women. *J. Natl Cancer Inst.*, **53**, 647-654.

SWANSON, C.A., BRINTON, L.A., TAYLOR, P.R., LICITRA, L.M., ZIEGLER, R.G. & SCHAIRER, C. (1989). Body size and breast cancer risk assessed in women participating in the breast cancer detection demonstration project. *Am. J. Epidemiol.*, **130**, 1133-1141.

TARTTER, P.I., PAPATESTAS, A.E., IOANNOVICH, J., MULVIHILL, M.N., LESNICK, G., AUFSES, Jr, A.H. (1981). Cholesterol and obesity as prognostic factors in breast cancer. *Cancer*, **47**, 2222-2227.

TORNBERG, S.A., HOLM, L.E. & CARSTENSEN, J.M. (1988). Breast cancer risk in relation to serum cholesterol, serum beta-lipoprotein, height, weight, and blood pressure. *Acta Oncol.* **27**, 31-37.

TRETLI, S., HALDORSEN, T. & OTTESTAD, L. (1990). The effect of pre-morbid height and weight on the survival of breast cancer patients. *Br. J. Cancer*, **62**, 299-303.

VALAORAS, V.G., MACMAHON, B., TRICHOPOULOS, D. & POLYCHRONOPOULOU, A. (1969). Lactation and reproductive histories of breast cancer patients in greater Athens. *Int. J. Cancer*, **4**, 350-363.

WEBER, D.J., RUTALA, W.A., SAMSA, G.P., SANTIMAW, J.E. & LEMON, S.M. (1985). Obesity as a predictor of poor antibody response to hepatitis B vaccine. *JAMA*, **254**, 3187-3189.

WEBER, D.J., RUTALA, W.A., SAMSA, G.P., BRADSHAW, S.E. & LEMON, S.M. (1987). Impaired immunogenicity of hepatitis B vaccine in obese persons. *N. Engl. J. Med.*, **314**, 1393.

WYNDER, E.L. & COHEN, L.A. (1982). A rationale for dietary intervention in the treatment of postmenopausal breast cancer patients. *Nutr. Cancer*, **3**, 195-199.

WYNDER, E.L., MACCORNACK, F.A. & STELLMAN, S.D. (1978). The epidemiology of breast cancer in 785 United States Caucasian women. *Cancer*, **41**, 2341-2354.

WYNDER, E.L., MORABIA, A., ROSE, D.P. & COHEN, L.A. (1990). Clinical trials of dietary interventions to enhance cancer survival. In *Recent Progress in Research on Nutrition and Cancer*, Mettlin, C.J. & Aoki, K. (eds) pp. 217-229. Wiley-Liss: New York.

ZUMOFF, B. & DASGUPTA, I. (1983). Relationship between body weight and the incidence of positive axillary nodes at mastectomy for breast cancer. *J. Surg. Oncol.*, **22**, 217-220.